# Comparing circular and network buffers to examine the influence of land use on walking for leisure and errands

**DOI:** 10.1186/1476-072X-6-41

**Published:** 2007-09-20

**Authors:** Lisa N Oliver, Nadine Schuurman, Alexander W Hall

**Affiliations:** 1 Department of Geography, Simon Fraser University, Burnaby, British Columbia, Canada

## Abstract

**Background:**

There is increasing interest in examining the influence of the built environment on physical activity. High-resolution data in a geographic information system is increasingly being used to measure salient aspects of the built environment and studies often use circular or road network buffers to measure land use around an individual's home address. However, little research has examined the extent to which the selection of circular or road network buffers influences the results of analysis.

The objective of this study is to examine the influence of land use type (residential, commercial, recreational and park land and institutional land) on 'walking for leisure' and 'walking for errands' using 1 km circular and line-based road network buffers. Data on individual walking patterns is obtained from a survey of 1311 respondents in greater Vancouver and respondent's postal code centroids were used to construct the individual buffers. Logistic regression was used for statistical analysis.

**Results:**

Using line-based road network buffers, increasing proportion of institutional land significantly reduced the odds of 'walking for leisure 15 minutes or less per day' no significant results were found for circular buffers. A greater proportion of residential land significantly increased the odds of 'walking for errands less than 1 hour per week' for line-based road network buffer while no significant results for circular buffers. An increased proportion of commercial land significantly decreased the odds of 'walking for errands less than 1 hour per week' for both circular and line-based road network buffers.

**Conclusion:**

The selection of network or circular buffers has a considerable influence on the results of analysis. Land use characteristics generally show greater associations with walking using line-based road network buffers than circular buffers. These results show that researchers need to carefully consider the most appropriate buffer with which to calculate land use characteristics.

## Background

Rising obesity rates in Canada is an important public health problem because of co-morbidities such as heart disease, diabetes and cancer [1]. Increasing rates of obesity are linked to a changing social and physical environment rather than genetic factors [2] and research has begun to investigate the role of the built environment in influencing obesity by promoting or inhibiting physical activity [3-5]. Walking is important from a public health perspective because it is the most common physical activity among Canadians [6]. While Canada's Physical Activity Guide [7] recommends Canadians accumulate at least 30 minutes of moderate physical activity (e.g. walking) per day a national survey has demonstrated that only 49% of Canadians meet this requirement [6].

An increasing number of studies demonstrate that aspects of the built environment can both promote and discourage walking [4,5,8-11]. Common measures of the built environment include land use type, density (e.g. residential density), land use mix and street connectivity (e.g. intersections per km^2^) [3].

A recent study of US metropolitan areas has demonstrated that a greater diversity of businesses in a neighbourhood increases walking [12]. Greater proximity to shopping centres is associated with increased walking in older adults [13]. Studies examining the influence of park land and green space on walking have demonstrated mixed results. In a study of 56 neighbourhoods in Portland a positive relationship between green space and physical activity among older adults was found [14]. A study of women in Melbourne, Australia demonstrated that public open space (e.g. parks) did not influence walking for leisure or transport among women [15]. Giles-Corti et al [16] examined 1,802 adults in Perth, Australia and results showed that open space was positively related to walking for transport but not walking for recreation. Residential density has been positively associated with walking in several studies [14,17]. Measures of the built environment using indices (e.g. land use mix) have been associated with walking in several studies [14,18]. Frank et al [8] in a study of adults in Atlanta has found more walkable neighbourhoods, measured using land use mix, residential density and intersection density, were associated with increased physical activity. More walkable neighbourhoods have also been associated with increased walking among older adults [19].

Research examining the influence of the built environment on physical activity and health has been limited by two factors. First, there is a need for theories or conceptual models articulating how aspects of the built environment may influence physical activity or other health outcomes [20-23]. Specifying conceptual models is important to identify and to understand how salient aspects of the built environment influence walking [22]. Walking is influenced by physical characteristics such as the variety of potential destinations as well as aesthetics of the built environment [22]. Second, limitations in spatial epidemiology, geographic information systems (GIS) and geographic data have made it difficult to empirically test models and hypotheses. Until recently most studies have relied on perceived rather than objective measures of the built environment [23]. However, recent advances have resulted in the ability to identify the spatial location of respondents and use high-resolution spatial data to objectively measure their local environments [3,5,8,23]. High-resolution road network data, land use data, cadastral data and census data are increasingly available to measure aspects of the built environment that may influence walking. This use of objective measures of the built environment is important to better understand how land use may influence walking behaviours and also to identify specific dimensions that could be used by planners and policy makers to increase walking [23]. Increasing availability of high-resolution spatial data combined with the computational power of GIS means that many options are available to measure aspects of the built environment that influence walking; however, there has been relatively little focus on developing and testing methodologies to measure the built environment.

To assess relations between the local built environment and physical activity it is necessary to define a spatial unit that best represents a respondent's local environment. The characteristics of this local environment, such as aesthetics and built environment factors are presumed to influence an individual's decision to engage in physical activity. There is a dearth of research examining the most appropriate method to define spatial units or assessing the sensitivity of results to the choice of spatial unit. Typically, one of three methods is used to define spatial units to measure aspects of the built environment. One method is to use pre-defined spatial units such as a census tracts or planning neighbourhoods to construct measures of the built environment [12,23,24]. A problem with using predefined areas is they do not necessarily correspond to areas an individual may walk. Built environment measures based on these units may have less error for individuals living in the centre of the unit than the edges potentially introducing bias into analysis. Given shortcomings with this technique, some studies have begun to use locational information (e.g. addresses, postal codes) to define unique areas for each individual. The most common method has been to establish a circular buffer around respondent's geocoded location at a given radius [19,25-27]. While likely providing a more representative assessment of the built environment that may influence walking, a shortcoming is that a circle may not accurately represent the spatial area that influences walking. Circular buffers are likely to be inaccurate in areas with natural features such as rivers, lakes and cliffs or built features such as railways or suburbs with poor street connectivity. In such cases, areas within the buffer may be inaccessible by the respondent but still used to calculate built environment measures.

Because of the limitations of circular buffers, a few studies have used road network buffers to define areas within which an individual can walk as a base for measuring the built environment. Some studies have calculated distance to recreational facilities using ArcGIS network analyst to determine the shortest distance from the respondents' home by road [14,28]. A polygon-based road network buffer has been used to define one-kilometre (km) areas around respondents' home locations [8]. In this method, the endpoints of all possible journeys up to 1 km along the road network from the individual respondent are used to form the vertices of an irregular polygon that defines the traversable area within 1 km of the respondent's location. While this method may provide a more accurate assessment of the actual land area that influences walking, joining road vertices using straight lines may lead to inaccuracies in areas that do not have dense, regular grid street patterns.

**Figure 1:**
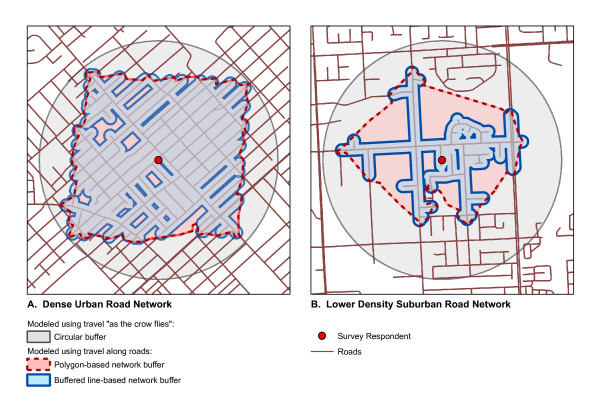
**Comparison of buffer methods for assessing neighbourhood land use for dense and suburban road networks**. The circular buffer method includes all land up to 1 km from the individual "as the crow flies" ('circular method', dark gray). This buffer fails to account for how the existing road network restricts the manner in which an individual is able to traverse the landscape. The other two approaches both consider how the road network restricts travel, affecting what is actually accessible within 1 km of travel. The polygon-based network buffer ('polygon method', red) uses the end points of 1 km journeys in the network as the vertices with which to construct an irregular polygon to define the accessible "neighbourhood". The method presented in this paper defines the 1 km neighbourhood by applying a 50 m buffer to a 950 m line-based network buffer ('buffered line method', blue), thus more closely approximating the roads accessible to the individual. The difference between the methods is related to the street pattern. For grid road networks (high connectivity) (A), the difference between the circular method and the network-based methods is moderate with the latter offering only slight improvements in the representation of a "local neighbourhood". However, for irregular road networks (lower connectivity) in suburban settings (B), two important changes are observed. Firstly, the circular method becomes a much less useful approximation compared to those that account for the structure of the road network. Secondly, there is a substantial difference between the polygon method and the buffered line method.

**Figure 2:**
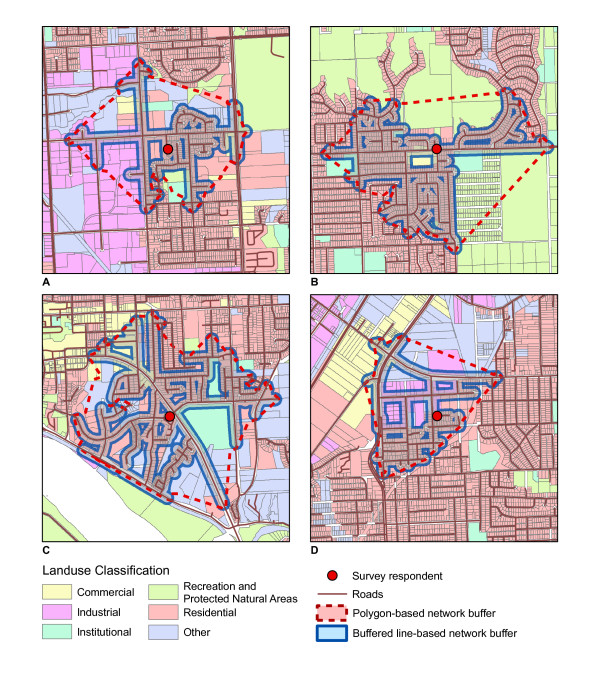
**Comparison of network buffer methods to evaluation of local neighbourhood land use composition**. Four examples below compare the buffered line-based network buffer ('buffered line method') to the polygon-based network buffer ('polygon method'). (A) The use of the polygon method adds a substantial amount of area to the local neighbourhood that is not actually accessible by an individual. (B) Using the buffered line method, it is evident that this individual would experienced his/her neighbourhood as being overwhelmingly residential but the polygon method would understate this experience by adding two large areas that cannot be meaningfully interacted with. In particular the large region to north of this individual appears to be completely concealed from the nearest roads by the houses that line the streets – its inclusion in the polygon method will greatly overestimate the presence of green space relative to a model focused on where the individual can actually cover walking. (C) The percentages of both institutional and 'other' land are greater when using the polygon method, thereby decreasing the relative weight given to land more practically accessible. (D) The polygon method may overstate the industrial land's importance within this individual's local neighbourhood, in terms of the influence on walking.

No studies we are aware of have measured land use alongside roads, which may better assess of aspects of the built environment that influence walking. In urban and suburban areas where the majority of land is privately owned, walking typically takes place on public sidewalks or along roads. This type of approach may also provide results that are more valid in areas with non-grid street networks. Figure 1 shows a comparison of a circular buffer, polygon based network buffer and line-based road network buffer for a dense urban road network and a lower density suburban road network. The polygon based network buffer and line-based network buffer are similar for the dense road network but quite different for the suburban road network. Using a line-based buffer approach may also provide results that are less sensitive to the presence of a single land use skewing results. In this approach a buffer of a specified width is placed around a line-based road network buffer. Because studies examining the influence of land use on walking typically use small buffers (e.g. 1 km) it is possible for a single land use or parcel of land (e.g. school, park, or shopping mall) to provide a skewed measure of the built environment. Figure 2 compares polygon based network buffers and buffered line-based road network buffers for neighbourhoods with different types of land use and demonstrates that polygon based buffers may be skewed in areas with large parcels of industrial or park land. Using network buffers is a new area of research and has the potential to more accurately define areas of the built environment that influence walking. Road network buffers are commonly used in applications such as hospital travel times; however, comparably little research has examined the most appropriate method to construct network buffers to examine relations between land use and physical activity [29]. Constructing a road network buffers is considerably more complex and computationally intensive than circular buffers and research has not evaluated if results are improved using a road network buffer.

The present study was designed to fill two gaps in this research area. The first goal of this paper is to present a new methodology to construct road-based network buffer in a geographic information system that may more accurately represent the 'walkable' neighbourhood of an individual and capture areas of an individual's local environment that may most directly influence walking. The second goal is to assess the extent to which the selection of circular or road-based network buffers affects the results of analysis examining the influence of land use on walking patterns. This paper is unique in that it specifically compares network and circular buffers and as such is an important addition to research examining the influence of the built environment on walking and physical activity.

The paper is divided into three remaining sections. The methods section discusses the individual data used, construction of circular and line-based road network buffers, measurement of the built environment and analysis methods. The results section presents the results of the analysis. The final section of the paper discusses the implications of the analysis and areas for future research.

## Methods

### Individual data

Individual data was collected using a telephone survey in eight neighbourhood clusters in suburban municipalities (i.e. outside of the City of Vancouver) of the Greater Vancouver Regional District. The areas were created by clustering three to four census tracts, which are small geographic areas defined by Statistics Canada with a population between 2,500 and 8,000. The population of the neighbourhood clusters ranged from 11,000 to 18,000 (2001 Census of Canada). Areas were selected based on their median family income (2001 Census of Canada) and residential density (population per hectares of residential land). Residential density and median family income was determined for all Census Tracts in the Greater Vancouver Regional District. Residential density was selected because it is associated with walking and is correlated other dimensions of the built environment such as land use mix and connectivity [8]. The aim was to select areas with equivalent income levels but differing residential densities. Four clusters with mid-high residential density (70 to 122 people per hectare of residential land) were selected, two of which with mid-high median family income ($53,000 – $77,000 CDN) and two of which with mid-low median family income ($32,000 – $44,000 CDN). Four clusters with mid-low residential density (29 to 61 people per hectare of residential land) were also selected, two of which with mid-high median family income and two of which with mid-low median family income.

A sampling frame of households for each neighbourhood was generated from the local telephone provider and numbers were de-duped to remove multiple (i.e. 2 lines per household), ineligible (e.g. fax, business) or invalid numbers. Random Digit Dialling (RDD) was used to select a household from the sampling frame and a minimum of five call-backs were made to reduce bias due to non-response. Interviews were conducted by experienced telephone interviewers using Computer Assisted Telephone Interviewing (CATI). The survey was piloted in January 2006 and the full survey was conducted over two-weeks in February 2006. Data was collected for 1935 adults and the survey achieved a response rate of 29% calculated as the percent of co-operative contacts divided by the number of contacts. In this current study we use data for respondents between 20 and 60 years of age. There were 1529 respondents in this age range and 218 were excluded due to missing variables or an invalid postal code resulting in an analytic sample of 1311.

### Dependent Variables

The survey assessed the amount of time respondents spend walking both for errands and for leisure. These were selected as dependent variables because they may be associated with different land uses (e.g. walking for leisure may be associated with park land and walking for errands may be associated with commercial land) and as such are a useful way to examine associations with measures of the built environment. Walking for errands was assessed using the item "In a typical week in the past few months how many hours did you spend walking from home to grocery stores, banks, or to do other errands? none, less than 1 hour, from 1 to 5 hours, from 6 to 10 hours, from 11 to 20 hours, greater than 20". For analysis a dichotomous variable was constructed from the responses (less than one hour = 1, one hour or greater = 0). Walking for leisure was assessed used the item "On a typical day in the past 3 months, how much time did you spend walking for leisure? 0 minutes, 15 minutes or less, 16 – 30 minutes, 31 minutes to one hour, over an hour." The responses were dichotomized for analysis (15 minutes or less = 1, greater than 15 minutes = 0). Canada's Physical Activity Guide recommends adults engage in at least 30 minutes of physical activity (e.g. walking, house cleaning, weightlifting, running) to be accumulated in 10 minute intervals [7]. The cut-off of 'greater than 15 minutes' was selected because individuals walking this amount would meet at least half of their daily physical activity requirements through walking for leisure.

### Independent Variables

Five demographic variables were included to control for potential confounding with the outcome variables. Gender (female = 1, male = 0) and age (continuous) were included in all models. Household income was based on respondent self-report and three categories were created: low income (less than $40,000 CDN) middle income ($40,000 to $80,000 CDN) and high income ($80,000 CDN and over). Dummy variables were created and middle income was the reference category. Respondent's marital status was categorized into single, married/common law and divorced/widowed. Dummy variables were constructed and married/common law was the reference category. Body Mass Index (BMI, weight (kg)/height (m)^2^) was calculated based on self-reported heights and weights. This variable was included because a higher BMI may be independently associated with lower levels of physical activity. The presence of a self-reported chronic condition (yes = 1, no = 0) was included because such conditions may limit respondents ability to engage in walking activities.

### Creation of circular and line-based road network buffers

Four steps were involved in the creation of the circular and line-based road network buffers. First, survey respondents were geocoded using the Statistics Canada Postal Code Conversion File to assign a latitude and longitude co-ordinate to the centroid of respondent's self-reported six digit postal code. Postal codes were used a proxy for respondents actual street address location. A study has found 88% of Canadians in urban areas have a postal code within 200 metres of their true address location [30].

The second step was the creation of both circular and line-based road network buffers around each respondent's postal code centroid. A circular buffer of 1 km radius was constructed around the centroid of each survey respondent's postal code in ArcGIS 9 from Environmental Systems Research Institute (ESRI). A buffered line-based road network buffer was created using the British Columbia Road Network file, from GIS Innovations, which includes all roads in the study area. ArcGIS network analyst was used to calculate a 950 metre line-based buffer along the road network from each respondent's postal code centroid. A 50 metre simple buffer was then constructed around this line-based buffer, resulting in a 1000 metre buffer (950 m along the road plus 50 m away from the road) around each individual constrained to contiguous roadway. Only the portion of parcels that were within 50 m of the roadway were included in calculations. This may represent a better approximation of potential destinations locally accessible to the individual respondent.

A 50 metre buffer was chosen to ensure that parcels along the selected roads would be included but that most parcels located further from the road (e.g. behind those adjacent to the road) would not be selected. This method is based on the idea that land use encountered along roads is most important in characterizing a neighbourhood in the way it is experienced by residents walking through it, and land not accessible to the pedestrian, even if physically nearby, is not part of their 1 km walking neighbourhood. A 50 metre buffer of the road generally appears to be well-suited to constructing such a model and ensures that everything along the road is captured, that deeply extended parcels are not overrepresented and that inaccessible land, for example behind the houses along the road, is excluded. A 100 metre buffer was too large as it often included such inaccessible parcels and could bias the weight of parcels whose depth away from the road is disproportionate with their "shop front" profile experienced by walkers along the road. A 25 metre buffer was too narrow as it would sometimes miss properties set slightly back from the road along wide roadways or wide right-of-ways.

The third step involved intersecting the circular and line-based road network buffer for each respondent with high-resolution land use data to calculate the local land-use composition for each individual as a measure of the local built environment. Greater Vancouver Land Use Data, which assigns every parcel of land in our study area a detailed code indicating the specific use of the property (e.g. city hall, sports facility), was obtained. For this analysis a simplified layer of five land use categories was created from the more detailed land use codes [31]. *Recreational and park land *includes parks, play grounds, fields, and trails/wooded areas. *Residential land *includes all private and rental dwellings such as high rises, low rises, garden/town homes, and single detached homes. *Commercial land *includes businesses with retail sales and services and professional offices. The category of mixed commercial and residential land (e.g. residential units above commercial properties) was divided between commercial and residential land uses. *Institutional land *includes public offices, hospitals, libraries, community centres, schools, city hall, and correction facilities. *Industrial land *includes factories, processing plant and industrial parks. An *other *land use category included water, open, undeveloped and agricultural land. The simplified land use layer was intersected with each respondent's circular and line-based road network buffer to create a layer including only land uses falling within the buffer area.

The total area (m^2^) of each of the five land use categories was calculated in ArcGIS for each respondent's circular and line-based road network buffer and then calculated as a proportion of the total area within each respondent's 1 km buffer. Land classified as 'other' was excluded from all further analyses. Proportions were chosen rather than an area measure of each land use category because the sizes of the line-based road network buffers were not equal among respondents. Even with the circular buffers, proportions were necessary as the total area of defined land use differs among respondents' buffers due to variations in the area of "empty" unclassified land occupied by streets and highways.

### Statistical Analysis

Logistic regression was used to examine the influence of the proportion of different land uses on the two outcome variables, walking for leisure and walking for errands. Parallel analyses were run using values for the proportion of land use categories as calculated by both the circular buffer and line-based road network buffer method, facilitating a comparison of the respective influence of each method of determining local land use composition for each respondent. A series of five models are presented beginning with a preliminary model (Model 1) that only includes individual characteristics. The following four models (Models 2–5) add each of the land use variables for both circular buffers (Model 2a-5a) and line-based road network buffers (Models 2b-5b). Model fit was assessed using the -2 Log Likelihood (-2LL) statistic, which indicates the amount of unexplained model variance. A lower -2LL statistic indicates a better model fit. The Likelihood Ratio Test (LRT) was used to determine if the addition of land use variables (i.e. Models 2ab-5ab) significantly improves the model fit compared to Model 1, which only includes individual variables. This LRT is calculated by subtracting the -2LL statistic for a model with land use characteristics from the preliminary model (e.g. Model 2a) and determining if this value exceeds the critical value of a chi-square distribution using a significance level of p = 0.05.

## Results

### Sample characteristics

The characteristics for survey respondents are presented in Table 1. For the two outcome variables, 30.76% of respondents spent 15 minutes or less per day walking for leisure and almost half of respondents spent less than one hour walking for errands per week. The age range of respondents in this study was from 20 to 60 years old and the average age of respondents was 42.52 years. The gender of respondents was somewhat skewed with 61.40% female and the average BMI was 25.72. The marital status of respondents was predominantly married or common law at 68.58%. The percentage of the sample in the lowest income (28.15%) and highest income (30.05%) categories was similar with a greater proportion in the middle income category (41.80%).

**Table 1:** Descriptive statistics for individuals aged 20–60 living in the Greater Vancouver Regional District (n = 1311)

Variable (N = 1311)	Percent	Average (SD*)
**Outcome**		
Walk for leisure 15 minutes or less per day	30.76%	--
Walk for errands less than one hour per week	49.11%	--
**Predictors**		
Age	--	42.52 (10.12)
Gender (Female)	61.40%	--
Body Mass Index	--	25.72 (5.66)
*Marital Status*		
Single	20.06%	--
Married/Common Law	68.58%	--
Divorced or widowed	11.36%	--
*Income*		
Less than $40,000	28.15%	--
$40,000 – $80,000	41.80%	--
More than $80,000	30.05%	--

*SD = Standard Deviation

### Comparison of circular and line-based road network buffers

Descriptive statistics for the circular and line-based road network buffers are presented in Table 2. The percent of commercial land was 7.35% for the circular buffer and increased slightly to 8.93% for the line-based road network buffer. The clustering of commercial businesses around roads is likely responsible for the increased percentage using line-based road network buffers since, being much more restricted to accessible roadways and truncating land situated in large gaps between roads, this method increases the weighting of roadside land parcels, more accurately representing the built environment as experienced by someone walking through it. Institutional land is similar for both buffer types. The percent of park and recreational land decreased from 11.81% for the circular buffers to 4.47% for the line-based road network buffers and this decrease is likely due to large areas of parkland falling beyond the 50 m road buffers. Residential land increased from 51.25% for the circular buffers to 64.94% for the line-based road network buffers. Residential properties typically do not extend much more than 50 metres back from the roads upon which they are located, meaning that the use of the line-based road network buffer excludes less residential land than other land uses such as industrial or agriculture occupying large land parcels.

**Table 2:** Characteristics of 1 km circular and network land use buffers for study participants

Land use type	Circular Buffer		Network Buffer	
	Percent	SD*	Percent	SD*
Commercial land	7.35%	6.84	8.93%	9.91
Institutional land	6.25%	4.65	5.14%	5.07
Recreational and park land	11.81%	6.65	4.47%	3.47
Residential land	51.25%	18.87	64.94%	24.15
Other land uses	17.14%	16.11	13.82%	20.45

*SD = Standard Deviation

### Logistic regression results

Logistic regression results for 'walking for leisure 15 minutes or less per day' are presented in Additional file 1. Model 1 includes only individual predictor variables with no land use characteristics. Being female significantly reduced the odds of 'walking for leisure 15 minutes or less per day'. Increasing BMI significantly increased the odds of 'walking for leisure 15 minutes or less per day' (OR 1.03; 95% CI 1.01,1.05). Income and marital status were not significant predictors of walking for leisure. Models 2 to 5 include individual and land use variables (added separately) for both the circular and line-based road network buffers. The -2LL is at the bottom of Additional file 1. Land use coefficients for Models 2, 3, 4 suggest recreational and park land, residential land and commercial land have no significant association with walking for leisure for both the circular and line-based road network buffers. The odds ratio for institutional land (OR 0.03, 95%CI 0.00, 0.33) was statistically significant for line-based road network buffers and indicates that increased institutional land reduces the odds of 'walking for leisure 15 minutes or less per day'. For institutional land (Model 5) the LRT indicates no significant difference between the individual model and circular model (Model 1 vs. Model 5a) but the line-based road network buffer significantly improves model fit (Model 1 vs. Model 5b).

Additional file 2 presents the model results for 'walking for errands less than one hour per week' and Model 1 presents the individual characteristics with no land use characteristics. Gender was not associated with walking for errands. Increasing BMI significantly increased the odds of walking less than one hour per week for errands and the magnitude of the odds ratios is similar to 'walking for leisure 15 minutes or less per day'. The odds of walking less than one hour per week for errands were significantly lower for individuals with low income (OR 0.65; 95% CI 0.49, 0.86) relative to middle income. Model 2 presents the results for recreational and park land for the circular and line-based road network buffers. For the line-based road network buffers increasing proportion of park land is associated with a decreased likelihood of walking for errands one hour or less per week. The LRT indicates that model fit was not improved by adding recreational land for the circular buffer but was significantly improved with the line-based road network buffer measure. Model 3 presents the results for residential land and shows that the proportion of residential land is not associated with walking for errands using the circular buffers but the line-based road network buffer shows increasing residential land significantly increases the likelihood that an individual will walk less than one hour per week for errands. Model 4 presents the results for commercial land and both buffers show that increasing commercial land significantly reduced the odds of walking less than one hour per week for leisure. The magnitude of the odds ratios were similar for both buffers (0.01 circular vs. 0.02 network). While the model fit, using the -2LL was significantly improved for both buffers compared to Model 1, the -2LL statistic was lower for the line-based road network buffer (1747.46 vs. 1755.77) suggesting that the line-based road network buffer may improve the model fit. Model 5 presents the results for institutional land use and results were only significant for the line-based road network buffers (OR 0.04; CI 95% 0.00, 0.35).

## Discussion

The principal goal of this paper was to present a methodology to create a line-based road network buffer that may better assess the land uses that influence walking. In this methodology a road network buffer that was 950 m long and 50 metres wide was constructed to assess aspects of land use that may influence walking. Previous methods to construct a network-based local neighbourhood have used journey endpoints (e.g. 1 km along the road network) as vertices from which to build a polygon delineating the neighbourhood. This method presents a vast improvement over the simple circular buffer method because it takes into account that the area of a "local neighbourhood" is necessarily restricted by the manner in which one is able to traverse the landscape – a pedestrian cannot travel "as the crow flies" but is instead forced to travel along existing roadways. However, the methodology currently presented even further improves the definition of a local neighbourhood by constraining the shape to more closely follow roads and thus exclude large inaccessible areas between diverging roads (see Figures 1 &2). This method may better represent the local neighbourhood of an individual as it would be experienced by that person as they walked through it. The advantage of the methodology presented is that it is less likely to be skewed by large features such as parks and industrial land as only land use within 50 metres of roads is selected. The methodology can be easily modified using GIS to meet the specific needs of other research projects. A shorter buffer length (e.g. 500 metres) may be more appropriate for studies with elderly populations or longer (e.g. 2 kms) for studies with younger populations. While we chose a buffer width of 50 metres, a wider or narrower may be more appropriate depending upon the built environment of the region under study.

The second purpose of this paper was to compare results of utilizing circular and line-based road network buffers to determine local neighbourhood land use composition, using walking for leisure and walking for errands as outcome variables. Greater association between land use and walking was found using the line-based road network buffers than the circular buffers suggesting that they may be better suited to examine relations between the built environment and walking. These results are important because they show that relations between the built environment and walking are sensitive to the choice of measure. For example, increasing residential land was associated with walking less for errands using line-based road network buffers but no association was found using circular buffers. The results of this study suggest that previous studies that have used circular buffers but found few associations with physical activity may see associations using line-based road network buffers. Based on these finding we suggest that when formulating policies to increase walking through modifying the built environment policy makers need to consider how the built environment was measured in different studies. Our results demonstrate that conclusions about which types of land use influence walking are sensitive to the type of buffer used.

Model results find that low income respondents, relative to middle income respondents, walk more for errands. Similarly, a study of walking behaviours of high and low socioeconomic adults found low socioeconomic status adults walk more for errands than high socioeconomic adults [32]. It is possible that lower income adults may walk more for errands due to reduced access to an automobile. Income was not significantly associated with walking for leisure. A study of women in Melbourne has also failed to find statistically significant differences in recreational walking by socio-economic status [16]. Increasing BMI was associated with an increased likelihood of walking less for both leisure and errands. These finding are supported by a national survey of Canadians which found that adults who are physically active in their leisure time are less likely to be obese [33].

In this study recreational land was not associated with walking for leisure. Some previous studies have similarly found no relations and others have demonstrated a positive relationship between recreational land and walking [14,16,34,35]. Future research should take into account the quality of park space and available facilities in the study areas. Increasing institutional land (e.g. libraries, hospitals, and community centres) reduced the odds of walking less for both leisure and errands (using line-based road network buffer) which was expected as institutional land serves both leisure and utilitarian functions. Increasing commercial land reduced the odds of walking less for errands and this was expected given similar findings in other studies [12,18].

This study has several strengths. First, the geographic areas in this study were pre-selected to have a range of residential densities and income levels. Spatially sampling diverse geographic areas is important to be able to examine relations between the built environment and health outcomes [36]. Second, in this study we used data on walking for leisure and walking for errands because these types of walking may be influenced by different land uses, therefore providing an opportunity to examine both network and circular buffers. Third, the availability of high-resolution land use data allowed us to measure aspects of the built environment in which respondents live.

There are several limitations to this study. The findings are based on cross-sectional data and therefore causality cannot be determined. Another limitation is that six digit postal codes were used as a proxy for respondent's home address and respondents who live in neighbourhood clusters with higher densities may have postal codes that are more accurate than those in lower density neighbourhood clusters. The survey was conducted in the month February, which is typically rainy and wet and the responses may be more conservative than if the survey was conducted in the summer. The survey item assessing walking for errands specifically asked about time spent walking from home to do errands; however the item assessing walking for leisure did not specify walking from home for leisure. In this study we only compared walking for leisure and walking for errands and other types of walking or physical activities were not examined but may also be related to aspects of the built environment. Comprehensive data on total daily physical activity was not available to enable to the construction of cut-off points indicating if respondents met Canada's Physical Activity Guidelines [7]. As such, cut-offs were selected to compare circular and network buffers and may have less utility from a public health perspective. Because we lacked information on where respondents walked we cannot confirm that the line-based road network buffer is a better approximation than the circular buffer of the local neighbourhood that influences walking. Line-based road network buffers were constructed on the assumption that walking occurs on sidewalks or along roads. Data was not available on the location of foot paths in parks or recreational areas where walking may also take place. The current research could be complemented by future studies that examine walking routes and how aspects of the built environment influence these routes. In this study aesthetics or the quality of the built environment was not assessed but may also influence walking behaviours. The data used was from a survey of eight neighbourhood clusters in suburban municipalities with contrasting income levels and residential density. As such, the respondents are not necessarily representative of the Greater Vancouver Regional District or Canadians.

In this study BMI was calculated using self-reported heights and weights which tend to underestimate BMI compared to direct measurements [37]. As the purpose of this study was to examine the influence of land use characteristics on walking using circular and line-based road network buffers we have not accounted for the full range of environmental factors such as social capital, safety, aesthetics, traffic flow, and quality of sidewalks that may influence walking [38]. In this study multilevel analysis, which is appropriate for the hierarchical data structure of individuals nested in eight neighbourhood clusters was not conducted due to an insufficient number of neighbourhood units [39]. The logistic regression models used were unable to account for the non-independence of observations nested within neighbourhoods [39].

## Conclusion

There is increasing interest in examining the influence of the built environment on walking, physical activity and obesity. The results of this study are important because they demonstrate that the selection of spatial units to measure the built environment influences the results of analysis. As high-resolution spatial data is increasingly being used to examine relations between the built environment and physical activity the results of this study demonstrate that researchers need to carefully consider the choice of spatial measure. For many models, greater associations between land use and walking were found using the line-based road network buffer suggesting that these buffers may be more sensitive than circular buffers to detect associations with walking. Existing studies finding no relations between the built environment and physical activity using circular buffers may find associations using line-based road network buffers. We suggest that when making conclusions about the influence of the built environment on physical activity researchers need to carefully consider the methodology used to measure the built environment. The results of this research highlight that future research is needed to develop appropriate measures of the built environment in order to better understand relations with physical activity.

## Competing interests

The author(s) declare that they have no competing interests.

## Authors' contributions

LNO conceived of the project and prepared the manuscript. NS developed the initial GIS methodology and AWH provided a novel adaptation of the GIS methodology. NS and AWH assisted with editing the manuscript. All authors have read and approved the final manuscript.

## Supplementary Material

Additional file 1 Logistic regression models predicting 'walking for leisure 15 minutes or less per day' by land use characteristics assessed with 1 km network and circular buffers. The data provided present the results of logistic regression models predicting 'walking for leisure 15 minutes or less per day' by land use characteristics assessed with 1 km network and circular buffers. Click here for file

Additional file 2 Logistic regression models predicting 'walking for errands less than 1 hour per week' by land use characteristics assessed with 1 km network and circular buffers. The data provided present the results of logistic regression models predicting 'walking for errands less than 1 hour per week' by land use characteristics assessed with 1 km network and circular buffers. Click here for file
