# Supplementary material for: Comparing circular and network buffers to examine the influence of land use on walking for leisure and errands
Source: Int J Health Geogr. 2007 Sep 20;6:41. doi: 10.1186/1476-072X-6-41 (PMC2034381; doi:10.1186/1476-072X-6-41)
Supplement: Additional file 2 — Logistic regression models predicting 'walking for errands less than 1 hour per week' by land use characteristics assessed with 1 km network and circular buffers. The data provided present the results of logistic regression models predicting 'walking for errands less than 1 hour per week' by land use characteristics assessed with 1 km network and circular buffers. [file 1476-072X-6-41-S2.pdf]

Additional file 2: Logistic regression models predicting 'walking for errands less than 1 hour per week' by land use characteristics assessed with 1km network and circular buffers

	Model 1		Model 2				Model 3			
	Individual characteristics		Recreational and park land				Residential land			
			(a) Circular Buffer		(b) Network Buffer		(a) Circular Buffer		(b) Network Buffer	
**Predictor variables**	**OR**	**95% CI**	**OR**	**95% CI**	**OR**	**95% CI**	**OR**	**95% CI**	**OR**	**95% CI**
Individual characteristics										
Age	1.00	(0.99,1.01)	1.00	(0.99,1.01)	1.00	(0.99,1.01)	1.00	(0.99,1.01)	1.00	(0.98,1.01)
Female (vs. male)	1.01	(0.80,1.27)	1.01	(0.80,1.27)	1.01	(0.80,1.28)	1.01	(0.80,1.28)	1.02	(0.81,1.29)
Chronic condition	0.85	(0.67,1.09)	0.85	(0.67,1.09)	0.86	(0.67,1.09)	0.87	(0.68,1.11)	0.88	(0.69,1.13)
Body Mass Index	1.02*	(1.00,1.05)	1.02*	(1.00,1.05)	1.03*	(1.00,1.05)	1.02*	(1.00,1.05)	1.03*	(1.00,1.05)
Low income (vs. mid income)	0.65*	(0.49,0.86)	0.65*	(0.49,0.86)	0.66*	(0.50,0.87)	0.64*	(0.48,0.85)	0.64*	(0.48,0.85)
High income (vs. mid income)	1.30	(0.99,1.69)	1.30	(0.99,1.70)	1.30*	(1.00,1.70)	1.30	(1.00,1.70)	1.28	(0.98,1.67)
Single (vs. married/common law)	0.85	(0.63,1.16)	0.85	(0.63,1.16)	0.86	(0.63,1.17)	0.85	(0.62,1.15)	0.86	(0.63,1.17)
Divorced/widowed (vs. married/common law)	0.97	(0.67,1.41)	0.97	(0.67,1.41)	0.99	(0.68,1.45)	0.98	(0.67,1.42)	1.01	(0.69,1.47)
Land use characteristics										
Park and recreational land %			0.89	(0.16,4.83)	0.02*	(0.00,0.47)				
Residential land %							1.55	(0.85,2.82)	2.46*	(1.53,3.96)
Commercial land %										
Institutional land %										
**Model Fit Statistic**										
-2 Log Likelihood	1782.10		1782.08		1776.16		1780.09		1768.22	

*p<0.05

Additional file 2: (cont'd) Logistic regression models predicting 'walking for errands less than 1 hour per week' by land use characteristics assessed with 1km network and circular buffers

	**Model 4**				**Model 5**			
	**Commercial land**				**Institutional land**			
	**(a) Circular Buffer**		**(b) Network Buffer**		**(a) Circular Buffer**		**(b) Network Buffer**	
**Predictor variables**	**OR**	**95% CI**	**OR**	**95% CI**	**OR**	**95% CI**	**OR**	**95% CI**
Individual characteristics								
Age	1.00	(0.98,1.01)	1.00	(0.98,1.01)	1.00	(0.99,1.01)	1.00	(0.99,1.01)
Female (vs. male)	0.97	(0.77,1.23)	0.98	(0.77,1.23)	1.02	(0.81,1.28)	1.02	(0.81,1.28)
Chronic condition	0.86	(0.67,1.10)	0.87	(0.67,1.10)	0.85	(0.67,1.09)	0.84	(0.66,1.08)
Body Mass Index	1.03*	(1.00,1.05)	1.03*	(1.00,1.05)	1.03*	(1.00,1.05)	1.03*	(1.00,1.05)
Low income (vs. mid income)	0.70*	(0.52,0.93)	0.71*	(0.52,0.93)	0.63*	(0.48,0.84)	0.62*	(0.47,0.83)
High income (vs. mid income)	1.16	(0.88,1.52)	1.19	(0.88,1.52)	1.31*	(1.00,1.71)	1.28	(0.98,1.67)
Single (vs. married/common law)	0.89	(0.65,1.22)	0.90	(0.65,1.22)	0.86	(0.63,1.16)	0.86	(0.63,1.17)
Divorced/widowed (vs. married/common law)	1.02	(0.70,1.49)	1.04	(0.70,1.49)	0.98	(0.68,1.42)	0.99	(0.68,1.44)
Land use characteristics								
Park and recreational land %								
Residential land %								
Commercial land %	0.01*	(0.00,0.06)	0.02*	(0.00,0.06)				
Institutional land %					0.12	(0.01,1.44)	0.04*	(0.00,0.35)
**Model Fit Statistic**								
-2 Log Likelihood	1755.77		1747.46		1779.28		1773.42	

*p<0.05
